# Factors That Affect the Mechanical Strength of Archaeological Wood—A Case Study of 18th-Century Wooden Water Pipes from Bóżnicza Street in Poznań, Poland

**DOI:** 10.3390/ma14247632

**Published:** 2021-12-11

**Authors:** Magdalena Broda, Carmen-Mihaela Popescu, Daniel Ilie Timpu, Dawid Rowiński, Edward Roszyk

**Affiliations:** 1 Department of Wood Science and Thermal Techniques, Faculty of Forestry and Wood Technology, Poznań University of Life Sciences, Wojska Polskiego 38/42, 60-637 Poznań, Poland; dawidrowinski@vp.pl (D.R.); edward.roszyk@up.poznan.pl (E.R.); 2 BioComposites Centre, Bangor University, Deiniol Road, Bangor LL57 2UW, Gwynedd, UK; 3 Petru Poni Institute of Macromolecular Chemistry of the Romanian Academy, 700487 Iasi, Romania; mihapop@icmpp.ro (C.-M.P.); dtimpu@icmpp.ro (D.I.T.); 4 Centre of Wood Science and Technology, Edinburgh Napier University, Edinburgh EH11 4EP, UK

**Keywords:** archaeological wood, water mains, mechanical properties, wood degradation, FT-IR, XRD, cellulose crystallinity, cellulose, infrared spectroscopy, compression strength

## Abstract

Large amounts of archaeological wood are often excavated during groundworks in cities and towns. Part of the unearthed artefacts is usually saved, conserved and then presented in museums. However, if the finding contains several similar objects, some of them could potentially be further employed for some other practical purposes. The research aimed to determine the mechanical performance of the remains of wooden water mains excavated at Bóżnicza street in Poznań, Poland and evaluate its potential usefulness for any practical purposes. First, wood density was determined along with its mechanical strength in compression. The density of archaeological wood identified as Scots pine was lower than contemporary pinewood (383 kg × m^−3^ vs. 572 kg × m^−3^); therefore, its mechanical properties in compression tests were also lower, as expected, making the wood unsuitable for any practical applications. However, the differences in modulus of elasticity and compressive strength were not justified by the differences in wood density. Further infrared spectroscopy and X-ray diffraction analyses revealed additional differences in chemical composition and cellulose crystallinity between archaeological and contemporary wood. The results indicated the decrease in carbohydrate content and cellulose crystallinity in degraded wood, which, in addition to wood density, apparently contribute to the deterioration in mechanical strength of archaeological wood. The case study of the excavated archaeological wooden pipes shows that they have historical value but are not useful for practical purposes. It also revealed that not only wood density but also its chemical composition and cellulose crystallinity level has a substantial impact on the wood mechanical properties, particularly in compression.

## 1. Introduction

The history of wooden water pipes dates back to the 17–19th centuries. They replaced stone, clay and terracotta water mains and aqueducts invented by ancient Greeks and Romans and, together with complex networks of pumps, water towers and reservoirs, they enabled the development of modern cities worldwide [1,2,3].

Usually, the wooden water main consisted of several sections about 1.8- to 3.6-m long bored logs with an internal diameter at an opening of 80 to 150 mm [4]. For early small-diameter pipes, the connections between sections were made by inserting a tapered end of one log into the broader end of the adjacent one and holding the joint together using an iron band. Sometimes bell-and-spigot joints made of lead were also applied [2,4]. Where large lines were required, wood-stave pipes made of narrow wood strips were also used [2]. To reinforce the structure of a wooden main, sometimes spiral wound wires or iron rods were installed on outside surfaces of the pipes [5].

Records about the existence of wooden water supply systems come from several different countries all around the world, including the United Kingdom (Liverpool—pipes made of beech or sycamore [4], London [2,3]), Japan (the Tatsumi Waterworks completed in 1632, the Kanda Waterworks [6]), USA (Leesburg in Virginia [7], Philadelphia [8,9], San Francisco [1], New York [10]), Ireland (Waterford—pipes made of red or white pine [11]), Finland (mostly pine) [12], Poland (Płock, Sieradz, Warszawa, Krosno, Poznań, Toruń, Kraków, Wiślica, Opatów, Przemyśl, Proszowice, Grudziądz, Bydgoszcz, Lublin; made mainly of coniferous wood, mostly pine, but also spruce or oak) [5,13,14]. In a few places, they are still in service [2,5].

Judging by the look of it, the excavated wood often seems to be relatively well-preserved [5,8,10,14]. Since often many similar artefacts are unearthed at the same place, part of them could potentially serve not only as museum objects, but also be used for some practical purposes. However, as a natural material, wood is prone to biodegradation and is usually degraded to some extent, which affects its performance, lowering use-values [15].

Wood buried in the soil deteriorates mainly due to the microbial activity of bacteria and fungi. Generally, in terrestrial environments, wood can be decomposed by the same wood-degrading bacteria that decompose waterlogged wood in aquatic environments, namely erosion, tunnelling, and cavitation bacteria. However, due to the high oxygen availability, the most pronounced wood decay in terrestrial ecosystems is caused by fungi. Based on the degradation pattern, fungal decay is categorised into three main types: white-, brown- and soft rot. White- and brown-rot fungi belong mainly to the Basidiomycota subdivision. White-rot fungi, in contrast with other organisms, can degrade all the cell wall components, including lignin. On the other hand, brown- and soft-rot fungi can depolymerise only cellulose and hemicelluloses, but they can also cause some modification in lignin. Overall, fungal decay can result in wood mass loss of even up to 97%. Therefore, we can rarely find wooden remains in terrestrial environments unless the burial conditions are deprived of oxygen and/or moisture necessary for microbial growth, preventing microbial degradation of wood tissue [16,17,18].

Wood mechanical properties depend on many different factors, starting with wood species, density and type of wood (juvenile, mature, reaction, early- or latewood) [19,20,21], through its hierarchical structure (including grain orientation and the orientation of microfibrils in different cell wall layers) [22,23,24,25,26] and the scale of measurements (a large board, small wooden block, cell wall, cell wall layer) [27,28,29,30], to the measurements conditions including the temperature and air relative humidity (reflecting in different wood moisture content) [24,31,32,33]. They are also determined by the composition and arrangement of the principal wood chemical components with cellulose microfibrils embedded in the hemicelluloses-lignin matrix [18,22,34,35,36,37].

Since the degradation processes change wood density and the composition of the main cell wall polymers, wood mechanical performance must also be affected [30,38,39,40,41].

**Figure 1:**
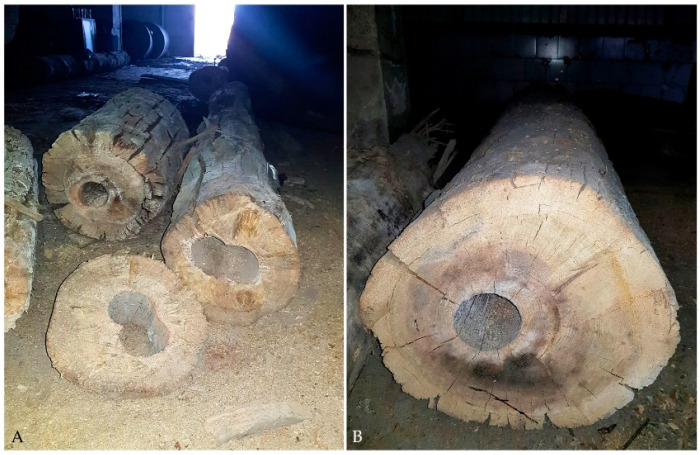
Elements of wooden water main excavated at Bóżnicza street in Poznań, Poland: (**A**) more degraded wooden pipes with deep cracks visible in the outer layers; (**B**) an example of a better-preserved log.

**Figure 2:**
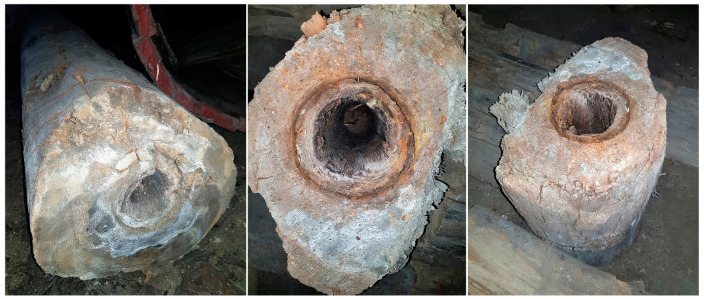
Degraded wooden elements of water pipes found during excavations at Bóżnicza street in Poznań, Poland.

In 2018, during the groundwork for a new building at Bóżnicza street in Poznań, Poland, large amounts of archaeological wood were unearthed. It was recognised as remains of wooden water mains of Poznań city dated back to the turn of the 18th and 19th centuries. Some of the excavated water pipes looked relatively good (Figure 1B), while others were more deteriorated (Figure 1A, Figure 2). In the light of this discovery, a question has been raised whether the archaeological wooden logs that undoubtedly provide us with a valuable picture of the ingenuity and skills of our ancestors should only serve as a witness of our history being publicly exhibited in the museum or maybe it could be employed for any other useful purposes. Whatever counterintuitive it may seem, it has been shown several times that selected mechanical properties of wood can remain unchanged or even improve slightly upon hundreds of years of ageing [42,43,44,45].

This research aimed to characterise the physico-mechanical properties of the excavated archaeological wooden pipes and evaluate their potential suitability for any other useful purposes. Therefore, except for identifying wood species and assessing its density, wood mechanical strength in compression was measured. Since the mechanical strength of archaeological wood turned out to be lower than what would result from its decreased density compared to contemporary sound wood of the same species, we decided to investigate what other factors affect the mechanical performance of the degraded wood. To achieve this, wood chemical composition was studied using Fourier-transform Infrared Spectroscopy along with the crystallinity of cellulose present in the cell wall using the X-ray diffraction method.

## 2. Materials and Methods

### 2.1. Materials

Archaeological wood used in the study comes from excavations at Bóżnicza street in Poznań, Poland. It was unearthed at the turn of 2018 and 2019. Part of the excavated wooden logs that were visually relatively well-preserved (Figure 1B) was used for the research.

Wood was identified as Scots pine (*Pinus sylvestris* L.—see Section 3.1). Therefore, for comparison, sound contemporary Scots pine wood from the Wielkopolska region, Poland, was also analysed.

### 2.2. Methods

**Figure 3:**
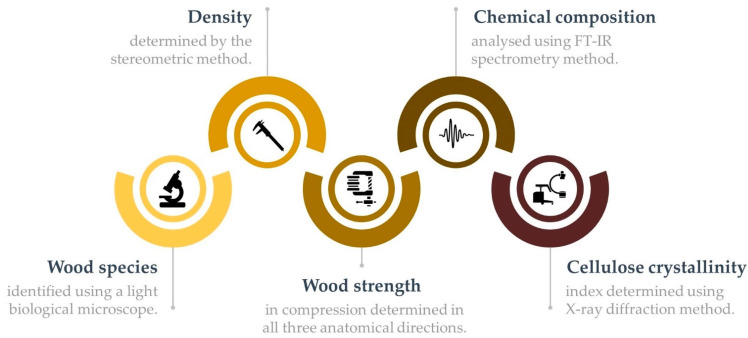
The course of experiments in the study on the mechanical properties of archaeological wooden water pipes.

To better understand the effects of archaeological wood degradation on its mechanical performance, a set of experiments were conducted, including the measurements of wood physical and chemical properties along with mechanical tests in all three anatomical directions (Figure 3).

#### 2.2.1. Wood Identification

Identification of the wood species of the excavated water pipes was made using light microscopy. First, small wood samples obtained from each wooden element were softened in a mixture of distilled water and glycerine (10% *v*/*v*). Then, they were sectioned in each anatomical direction with a microtome. Microscope slides were observed under a light biological microscope Motic B3 Professional Series (MoticEurope, S.L.U., Barcelona, Spain). Images of wood tissue were captured using a microscope-attached camera Moticam 2.0 (MoticEurope, S.L.U., Barcelona, Spain) coupled with a computer and analysed with a Motic Images Plus 2.0 (2017, MoticEurope, S.L.U., Barcelona, Spain) ML image analysis software.

#### 2.2.2. Sample Preparation and Density Measurements

Archaeological wooden log used in the study was selected among the excavated water pipes based on the ability to obtain a sufficient number of appropriately oriented and defectless wood samples required for mechanical tests.

Over 60 rectangular samples with dimensions 20 × 20 × 30 mm (in the radial, tangential and longitudinal directions, respectively) were cut out from the archaeological pine sapwood (a layer about 2–3 cm from the log perimeter). First, they were conditioned at room temperature (21 ± 1 °C) and ambient air relative humidity (40 ± 5%) until equilibrium moisture content was achieved. Then, the samples were weighed using an analytical balance accurate to 0.001 g (Sartorius GmbH, Göttingen, Germany). Next, their dimensions in the three principal anatomical directions were measured with a digital calliper accurate to 0.01 mm. Based on the results obtained, wood density was calculated as a ratio of the wood mass divided by its volume (according to ISO 13061-2:2014 [46]). After conditioning, average wood moisture content (based on 20 specimens) was determined using the standard oven-drying method (103 °C). It was calculated as a ratio between the mass of water contained in a seasoned sample to the mass of a dry sample. Finally, 30 specimens with the most similar density were selected for mechanical tests.

Similarly, contemporary pine sapwood samples with a macrostructure akin to archaeological wood were prepared.

#### 2.2.3. Compression Tests

Since the correlation between wood density and mechanical strength is the most pronounced in compression [47,48,49,50], we selected this type of mechanical testing. Due to wood degradation, it was impossible to obtain a sufficient number of normative samples for other mechanical tests, e.g., longer samples required for bending. Compression tests were performed using a numerically controlled Zwick Z050TH (Zwick/Roell, Ulm, Germany) universal testing machine. Samples of both wood types (archaeological and contemporary) were measured in all three anatomical directions (10 pieces per direction). For each specimen, two parameters were determined: the modulus of elasticity (MOE) and stress at proportionality limit (so-called compressive strength perpendicular to the grain or relative strength) or stress to failure (so-called compressive strength) for compression measurements in the tangential/radial or longitudinal direction, respectively.

#### 2.2.4. Infrared Spectroscopy

Fourier-Transform Infrared Spectroscopy (FT-IR) method was used to characterise wood chemical composition. Small pieces of all 10 replicates of archaeological and contemporary pine wood specimens were air-dried, powdered together (separately for archaeological and contemporary wood) and sieved. The wood powder fraction with an average particle diameter less than 0.2 mm was analysed with 4 cm^−1^ resolution using a Bruker ALPHA FT-IR spectrometer (Bruker, Billerica, MA, USA). Infrared spectra in the 4000–400 cm^−1^ region were recorded in KBr pellets. Wood sample concentration was 2 mg/200 mg KBr. Five spectra for each sample were recorded, and the resulting average spectrum obtained was processed using Grams 9.1 software (Thermo Fisher Scientific, Waltham, MA, USA).

#### 2.2.5. X-ray Diffraction

X-ray diffraction was applied to analyse cellulose crystallinity in archaeological and contemporary wood. Measurements were performed using a Diffractometer D8 ADVANCE (Bruker AXS, Berlin, Germany) with Cu-K radiation (λ = 0.1542 nm), a parallel beam with Gobel mirror and a Dynamic Scintillation detector. The working conditions during the measurements were 40 kV and 30 mA, 2 s/step, 0.02 degree/step. The processing of the diffractograms, i.e., their deconvolution, was performed using Grams 9.1 software (Thermo Fisher Scientific, Waltham, MA, USA). For the amorphous background, a Voight profile was used, while mixed Gaussian-Lorenzian profiles were applied for the crystalline regions. The crystallinity degree was calculated according to the equation proposed by Hermans and Weidinger [51]:

(1) $$Cr.I.\%=\frac{A_{cr}}{A_{t}}\times 100$$

where: *Cr.I.* % is the crystallinity degree, *A_cr_* is the crystalline area and *A_t_* is the total area under diffractogram.

## 3. Results and Discussion

### 3.1. Wood Identification

**Figure 4:**
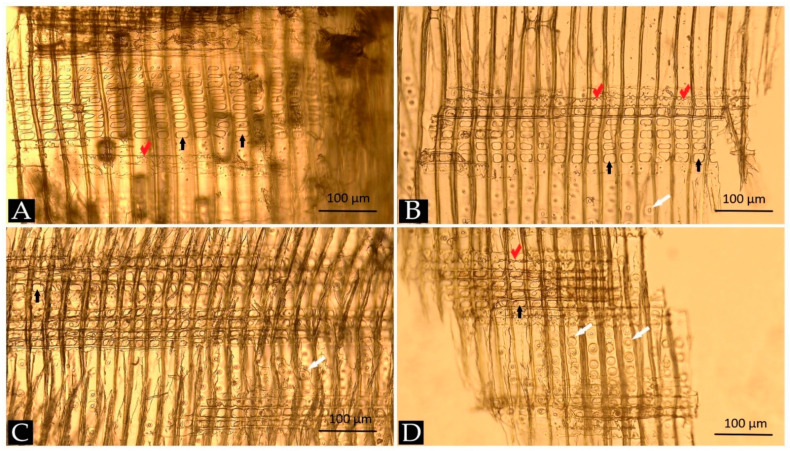
Images of radial sections (**A**–**D**) of archaeological wood from water pipes excavated at Bóżnicza street in Poznań showing anatomical details specific to pine species: fenestriform (window-like) pits—black arrows, bordered pits—white arrows, tooth-like projections (dentate) in ray tracheids—red ticks.

Wood identification was made based on its anatomy. The archaeological wood from water pipes was recognised as softwood; therefore, further species identification was based on radial sections. The most characteristic microscopic anatomical features that allowed recognising the wood as Scots pine (*Pinus sylvestris* L.) are presented in Figure 4.

All radial sections (Figure 4A–D) show horizontal heterocellular rays consisting of a few rows of ray parenchyma cells. They have large individual or twin fenestriform (window-like) pits (black arrows) where the parenchyma cells interconnect with vertical tracheids. Adjacent to parenchyma from both sides, the rows of horizontal ray tracheids can be seen with distinctive tooth-like projections on their cell walls (so-called dentate walls; red ticks in Figure 4A,B,D). Above and below rays, the vertical rows of tracheids are visible with single lines of small circular bordered pits (white arrows in Figure 4B–D). The anatomical details are specific to several pine species, including *P. sylvestris*, *P. mugo* and *P. nigra*. However, considering the geographical location and historical context of the water pipes excavation and the species range, the archaeological wood was identified as *Pinus sylvestris* [52,53].

### 3.2. Wood Density and Mechanical Properties

The physical properties of archaeological and contemporary pinewood are presented in Table 1. The average density value for contemporary pine is relatively high; however, it is still within average parameters for these species identified as 440–570 kg × m^−3^ according to Kollmann and Côté [20]. The density of archaeological wood from water pipes represents 67% of the contemporary pine density. It is also about 13% lower than the lower limit of medium pinewood density values. Considering similarities in the macrostructure between archaeological and contemporary pinewood, the density values obtained point to the degradation of excavated wood that has remained in the ground for over 200 years.

Measured after seasoning before the compression test, the wood moisture content of archaeological wood is slightly higher than contemporary sound wood (Table 1). Usually, the more degraded wood is, the higher its hygroscopicity is, which translates into higher moisture content compared to sound wood under the same conditions [54,55,56]. It results from the degradation of wood substance, leading to an increase in wood porosity and changes in the arrangement of the cell wall polymers. The loosened structure of the polymers facilitates the interactions of their hydroxyl groups with water molecules [56,57]. Increased porosity of the degraded wood reflects in its density reduction. This, in turn, affects wood mechanical properties so that a strong positive relationship between wood density and mechanical parameters such as modulus of elasticity, modulus of rupture or compressive strength exists [20,49,58,59].

The MOE values obtained in compression tests for archaeological wood with a density of 383 kg × m^−3^ and about 9% MC, MOE in the tangential (T), radial (R) and longitudinal (L) direction are 159, 189 and 4407 MPa, respectively (Table 1). In turn, the MOE values for sound contemporary pinewood with a density of 572 kg × m^−3^ and similar MC are 2.4, 4.2, and 4.8 times higher than degraded archaeological wood in the L, T, R direction. In both cases, the MOE values reflect the orthotropic nature of wood and show a typical order MOE_L_>>MOE_R_≥MOE_T_ [20,60].

Considering the differences in wood density between archaeological and contemporary wood, the lower MOE values for the former seem obvious. Therefore, we decided to relate MOE to wood density and calculate the specific modulus of elasticity (sMOE) as the value of the elastic modulus measured divided by the wood density. The calculated sMOE values for both wood types are presented in Table 1. They are 1.7 (in L direction) and 3 (in R and T directions) times higher for contemporary than for archaeological wood. The differences in the MOE and sMOE ratios of contemporary and archaeological wood allow the conclusion that the mechanical parameters measured do not depend solely on the wood density.

Apart from MOE, the relative/compressive strength (R_c_) was also measured to acquire a better picture of changes in the mechanical properties of the degraded wood (Table 2). Similarly to MOE, the R_c_ values show a typical order dependent on the anatomical direction (R_cL_ >> R_cR_ ≥ R_cT_). For contemporary pine, they are within the range of compressive strength specific for the species [61], and they are higher than archaeological wood (2.2, 3.5, and 3.3 times in L, T and R, respectively). Interestingly, when comparing the specific relative/compressive strength (sR_c_) values (calculated as R_c_ divided by the wood density) for contemporary and archaeological samples, they are only 1.5, 2.3, and 2.0 times higher. Different R_c_ and sR_c_ ratios for sound contemporary and degraded archaeological pine are in line with observed MOE and sMOE ratios. These indicate that not only wood density but also some other factors must have affected the mechanical properties of the degraded wood.

The results presented above prove that the long-term burial of wood in the soil and its functioning as a water supply system must have produced other changes in its structure than just the loss of wood substance manifested by its decreased density.

The wood mechanical strength strongly depends on its density. However, it also depends on the chemical composition and arrangement of the main polymers of the cell wall composed of cellulose microfibrils embedded in the hemicelluloses-lignin matrix [18,22,34,36]. Crystalline cellulose is mainly responsible for tensile strength. In contrast, amorphous cellulose, hemicelluloses and lignin contribute to the wood viscoelastic properties and determine its compression and hardness [18,61,62,63,64,65]. Since the degradation processes affect the composition and structure of the primary wood polymers, therefore, in the next step of the research, wood chemical composition was analysed together with the cellulose crystallinity index.

### 3.3. Wood Chemical Composition

**Figure 5:**
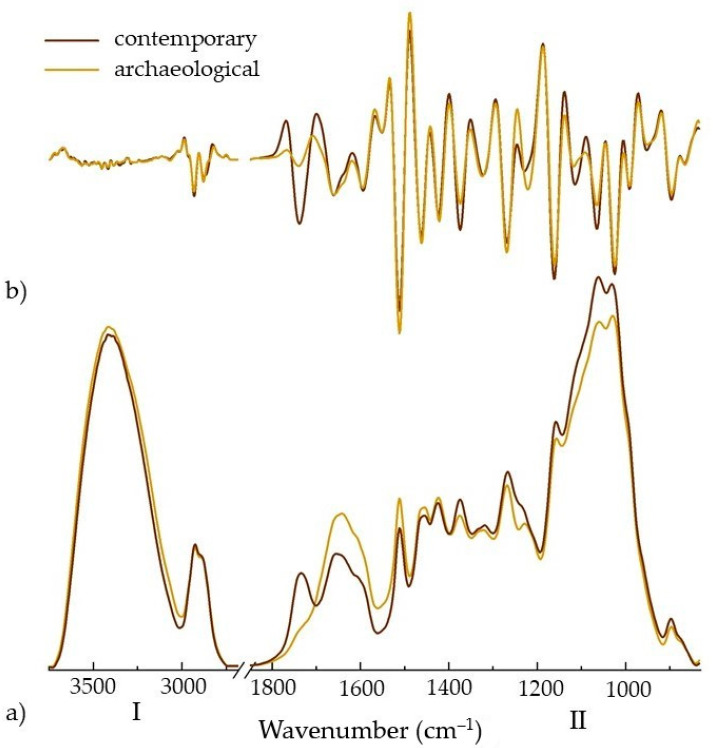
Infrared spectra (**a**) and their second derivatives (**b**) of sound contemporary and degraded archaeological pine wood.

The infrared spectra of the archaeological and contemporary pine wood samples are presented in Figure 5a. They show the typical wood spectrum shape with two regions, namely: (I) 3700–2700 cm^−1^ assigned to different inter- and intra-molecular hydrogen bonds as well as to -OH groups and methyl and methylene groups, and (II, so-called fingerprint region) 1850–830 cm^−1^ assigned to different specific stretching and deformation vibrations from all wood components. Since the bands in the wood spectra are generally overlapped, the second derivative will usually give detailed information about the modifications in its structure during ageing. Figure 5b presents the second derivatives of the spectra. The individual bands were identified, and their exact positions, as well as their assignments, are shown in Table 3.

As can be observed from both spectra and their derivatives, principal differences occur in the fingerprint region. The band from 1740/1741 cm^−1^ assigned to C=O stretching vibration of carboxyl and acetyl groups decreases in intensity in the archaeological wood spectrum, compared to the contemporary one. The same behaviour was observed for the bands assigned to C–H deformation in cellulose and hemicellulose, C–O–C vibration in cellulose and hemicellulose, and C–O stretching mainly from C(3)–O(3)H in cellulose I at 1375, 1161, 1115 and 1065 cm^−1^. At the same time, aside from a decrease in intensity, shifting to lower wavenumber was observed for the band at 1228 cm^−1^ (assigned to C–O–C stretching mode of the pyranose ring). A slight increase in intensity can be seen for the bands from 1640, 1512, 1461, 1421 and 1268 cm^−1^, assigned to absorbed O–H, C=C of aromatic skeletal (lignin), C–H deformation in lignin and carbohydrates and C–H bending mode in cellulose and C–O stretch in lignin.

The decrease in intensity of the band assigned mainly to carbohydrates, combined with the increase in the intensity of the water band and of the bands assigned mainly to lignin, indicate the higher degradation of the carbohydrates with lower degradation of lignin. Moreover, higher intensity of the water band suggests that archaeological wood presents a more hydrophilic structure than contemporary wood.

These observations—decreased concentrations of carbohydrates—indicate wood degradation. Moreover, they are well correlated with lower density usually observed for archaeological wood compared to the contemporary one since hemicelluloses and cellulose are the most available substrates for fungi and bacteria [72,73,74].

### 3.4. Cellulose Crystallinity

X-ray diffraction is mainly used to evaluate the degree of crystallinity in lignocellulosic materials. Among wood components, only cellulose shows a certain degree of crystallinity. Its regular structure and the presence of free hydroxyl groups that can be involved in different intra- and intermolecular hydrogen bonds may give rise to various ordered crystalline arrangements [75,76].

The natural form of cellulose in the wood cell wall (cellulose I) presents a typical diffractogram pattern with a high-intensity diffraction peak at 2theta = 22.3° (200 crystallographic plane of cellulose I), three other lower intensity diffraction bands at about 2theta = 14.8° (101 crystallographic plane), 2theta = 16.8° (crystallographic plane) and 2theta = 20.0° (102 crystallographic plane), and an amorphous background with its maximum at about 2theta = 18.5–19.2° [69,77,78].

**Figure 6:**
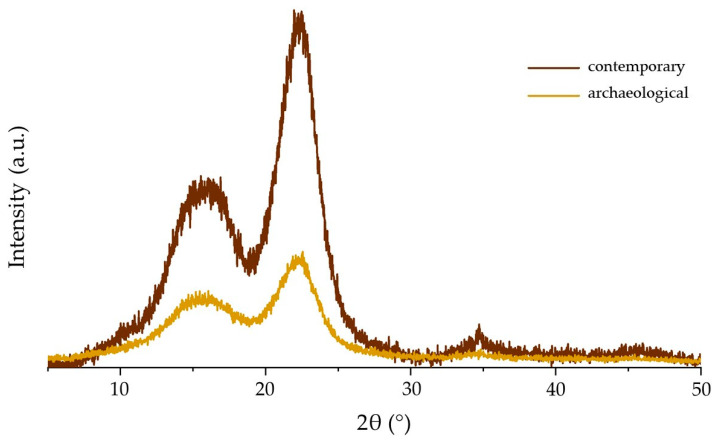
X-ray diffraction patterns of contemporary and archaeological pine wood samples.

The X-ray diffractograms of contemporary and archaeological pine wood are presented in Figure 6. They show typical peaks at about 2theta = 15.9/15.6° (which is an envelope of the individual peaks from 2theta = 14.8° and 16.8°) and 2theta = 22.23/22.10° (which is the envelope of the individual peaks from 2theta = 20.0° and 22.2°). The peaks for archaeological wood present lower intensities and larger width compared to contemporary pine wood diffractogram. The positions of the peaks maxima were slightly shifted to lower theta degrees in archaeological pine wood, indicating an increase in d-spacing resulting from wood degradation. The full width at half maximum of the diffraction peaks was higher for archaeological pine wood. It is known that the peak broadening may be due to a decrease in cellulose crystallite thickness, increase in packing defects, or compositional inhomogeneity and increased chain mobility of cellulose.

The determined crystallinity degree for archaeological pine wood was 42.5%. It was about 25% lower than the value obtained for contemporary pine wood (56.9%). The structural modification (indicating the degradation of carbohydrates) and the reduced crystallinity degree contribute to the observed reduction in mechanical properties of archaeological wood and explain the differences for specific modulus of elasticity and specific compressive strength between sound contemporary and degraded archaeological wood.

## 4. Conclusions

The mechanical analyses revealed that the elastic modulus in compression and compressive strength of archaeological wood is lower than expected from the decrease in its density compared to sound pine wood. Furthermore, FT-IR and XRD analyses showed changes in the chemical composition and structure of degraded wood, particularly the decrease in hemicelluloses and cellulose content and the reduced crystallinity degree, indicating the role of these components in the wood mechanical performance. Concluding, the case study shows that not only wood density but also its chemical composition and the crystallinity level has a substantial impact on the wood mechanical properties in compression. It also points out how both the physical and chemical properties of wood are crucial to understanding and predicting its performance. Moreover, it indicates how essential it is to characterise all the parameters to acquire a complete knowledge about the degraded wood artefact despite whether it is intended for conservation and exhibition in the museum or any other purposes.

In the light of the results obtained in the study, it is clear that wooden water pipes unearthed at Bóżnicza street in Poznań can serve mainly as historical artefacts. The degree of wood degradation reflected in its deteriorated mechanical properties makes the archaeological pinewood unsuitable for practical uses requiring mechanical strength. However, it could be used for decorative purposes that do not need to carry mechanical loads.

## Figures and Tables

**Table 1:** Density, actual moisture content (MC), modulus of elasticity (MOE) and specific modulus of elasticity (sMOE) in all principal anatomical directions (T—tangential, R—radial, L—longitudinal) for archaeological and contemporary pinewood.

Wood Type	Density (kg × m^−3^)	MC (%)	MOE_T_ (MPa)	sMOE_T_ × 10^6^ (N × m × kg^−1^)	MOE_R_ (MPa)	sMOE_R_ × 10^6^ (N×m×kg^−1^)	MOE_L_ (MPa)	sMOE_L_ × 10^6^ (N × m × kg^−1^)
Archaeological	383 ± 36	9.4 ± 0.2	159 ± 62	0.4 ± 0.1	189 ± 67	0.5 ± 0.2	4407 ± 1381	10.8 ± 2.6
Contemporary	572 ± 13	9.0 ± 0.2	662 ± 115	1.2 ± 0.2	909 ± 296	1.5 ± 0.5	10,775 ± 1572	18.7 ± 2.6

**Table 2:** Relative/compressive strength (R_c_) and specific relative/compressive strength (sR_c_) in all the principal anatomical directions (T—tangential, R—radial, L—longitudinal) for archaeological and contemporary pinewood.

Wood Type	R_cT_ (MPa)	sR_cT_ × 10^3^ (N × m × kg^−1^)	R_cR_ (MPa)	sR_cR_ ×10^3^ (N × m × kg^−1^)	R_cL_ (MPa)	sR_cL_ × 10^3^ (N × m × kg^−1^)
Archaeological	1.1 ± 0.6	2.9 ± 1.2	1.1 ± 0.3	3.0 ± 0.8	29.5 ± 5.6	72.6 ± 9.1
Contemporary	3.8 ± 0.4	6.7 ± 0.6	3.6 ± 0.4	6.2 ± 0.8	64.1 ± 3.9	111.3 ± 5.6

**Table 3:** Infrared bands positions and their assignments [66,67,68,69,70,71].

Bands Assignments	Bands Position	
	Contemporary Pine	Archaeological Pine
absorbed water weakly bound and intramolecular hydrogen bond in a phenolic group (in lignin)	3561	3564
O2–H2⋯O6 intramolecular stretching modes (in cellulose)	3417	3416
O5–H5⋯O3 intramolecular in cellulose	3349	3348
O6–H6⋯O3 intermolecular in cellulose I_β_ (3270)	3288	3288
O6–H6⋯O3 intermolecular in cellulose I_α_ (3240)	3221	3216
C–H stretching absorption in methyl and methylene groups in cellulose I	3135	3135
multiple formations of an intermolecular hydrogen bond between biphenol and other phenolic groups (in lignin)	3070	3067
multiple formations of an intermolecular hydrogen bond between biphenol and other phenolic groups (in lignin)	3012	3011
asymmetric C–H stretching	2931	2932
symmetric C–H stretching	2878	2878
C=O stretching vibration of carboxyl and acetyl groups	1740	1741
conjugated C–O in quinones	1660	1660
absorbed O–H	1638	1640
C=C of aromatic skeletal (lignin)	1594	1596
conjugated C–O	1551	1551
C=C of aromatic skeletal (lignin)	1512	1512
C–H deformation in lignin and carbohydrates	1462	1461
C–H deformation in lignin and carbohydrates	1423	1421
C–H deformation in cellulose and hemicellulose	1375	1374
C–H vibration in cellulose and Cl–O vibration in syringyl derivatives	1322	1325
C–H bending mode in cellulose and C–O stretch in lignin	1268	1268
C–O–C stretching mode of the pyranose ring	1228	1221
C–O–C vibration in cellulose and hemicellulose	1161	1161
C–O stretching	1115	1117
C–O stretching mainly from C(3)–O(3)H in cellulose I	1065	1065
C–O and C–C stretching ring in cellulose and hemicelluloses	1024	1024
C–O stretching	991	991
Aromatic C–H out of plane deformations, pyran ring stretching	952	952/936
C–H deformation in cellulose	896	896
CH out of plane vibrations in positions 2, 5 and 6 of guaiacyl units	866	866

## Data Availability

The data underlying this article will be shared upon reasonable request from the corresponding author.
